# Emerging Role of Epigenetics in Explaining Relationship of Periodontitis and Cardiovascular Diseases

**DOI:** 10.3390/diseases9030048

**Published:** 2021-06-29

**Authors:** Syed Ameer Hamza, Saba Asif, Zohaib Khurshid, Muhammad Sohail Zafar, Syed Akhtar Hussain Bokhari

**Affiliations:** 1 Department of Oral Medicine, University Medical & Dental College, Faisalabad 38000, Pakistan; syedameerhamza@yahoo.com; 2 Department of Periodontology, Sharif Medical & Dental College, Lahore 54000, Pakistan; sabba14@live.com; 3 Department of Prosthodontics and Dental Implantology, College of Dentistry, King Faisal University, Al-Ahsa 31982, Saudi Arabia; zsultan@kfu.edu.sa; 4 Department of Restorative Dentistry, College of Dentistry, Taibah University, Madinah Al Munawwrah 41311, Saudi Arabia; drsohail_78@hotmail.com; 5 Department of Dental Materials, Islamic International Dental College, Riphah International University, Islamabad 44000, Pakistan; 6 Department of Dental Public Health, College of Dentistry, King Faisal University, Al-Ahsa 31982, Saudi Arabia

**Keywords:** gum, genetics, epigenetics, periodontal diseases, cardiovascular diseases, diagnosis

## Abstract

Cardiovascular diseases such as ischemic heart diseases or stroke are among the leading cause of deaths globally, and evidence suggests that these diseases are modulated by a multifactorial and complex interplay of genetic, environmental, and lifestyle factors. Genetic predisposition and chronic exposure to modifiable risk factors have been explored to be involved in the pathophysiology of CVD. Environmental factors contribute to an individual’s propensity to develop major cardiovascular risk factors through epigenetic modifications of DNA and histones via miRNA regulation of protein translation that are types of epigenetic mechanisms and participate in disease development. Periodontal disease (PD) is one of the most common oral diseases in humans that is characterized by low-grade inflammation and has been shown to increase the risk of CVDs. Risk factors involved in PD and CVD are determined both genetically and behaviorally. Periodontal diseases such as chronic inflammation promote DNA methylation. Epigenetic modifications involved in the initiation and progression of atherosclerosis play an essential role in plaque development and vulnerability. Epigenetics has opened a new world to understand and manage human diseases, including CVDs and periodontal diseases. Genetic medicine has started a new era of epigenetics to overcome human diseases with various new methodology. Epigenetic profiling may aid in better diagnosis and stratification of patients showing potential predisposed states for disease. A better understanding of the exact regulatory mechanisms of epigenetic pathways driving inflammation is slowly emerging and will aid in developing novel tools for the treatment of disease.

## 1. Introduction

Cardiovascular diseases (CVD) are considered among the leading causes of deaths worldwide [1]. Genetic predisposition and chronic exposure to modifiable risk factors have been explored to be involved in the pathophysiology of CVDs [2,3]. Smoking, hypertension, alcohol, lack of exercise, insufficient consumption of fruits and vegetables, cholesterol, obesity, and diabetes are associated with CVDs [4]. The largest proportions of CVDs such as stroke and MI are attributed to metabolic risk factors with hypertension being the largest; however, other factors including tobacco, poor diet, low education, abdominal obesity, diabetes, physical activity, depression, and alcohol consumption each have relatively modest contributions to CVDs at a global level [5]. 

Periodontal disease (PD) among the most common oral diseases is well known to affect the world population [6] and is characterized by low-grade inflammation of tooth-supporting tissues [7]. Disease initiation and progression depends on a complex interaction of bacterial biofilm, pathogenic bacteria, and the host immune system of body [8]. Numerous modifying factors, including lifestyle, environmental factors, and genetics, are involved in the initiation and progression of this disease [9]. Periodontal disease has deleterious effects that are not restricted only to the oral cavity but extend beyond to other systems and oral inflammation that could promote loss of homeostasis at distant sites. [10]. As the research examining the systemic implications of periodontitis has grown exponentially in recent years [11], recent experimental studies have strengthened the potential causal link between periodontitis and its comorbidities by establishing a biologically plausible and clinically consistent mechanisms where periodontitis could initiate or aggravate a comorbid condition [12]. Both PD and CVD with high prevalence worldwide affect the social well-being of people [13]. Several conditions increase CVD risk, and it is revealed that CVD is mediated through infection or inflammatory pathway [14,15]. Oral inflammatory infections serve as metabolic stressors and may exacerbate systemic diseases [16]. Other studies confirm that periodontitis as an infectious and inflammatory condition increases risk of CVDs [14,17]. Periodontal disease is associated with local elevation of inflammatory cytokines (CRP, fibrinogen, haptoglobin, platelet-activating factor, IL-6, and IL-18) [18], and periodontal inflammatory process is accompanied by large network of cytokines and chemokines with high expression of proinflammatory cytokines such as interleukin (IL)-1α, IL-1β, IL-6, IL-12, tumor necrosis factor (TNF)-α, and regulatory cytokines such as IL-4, IL-1(RA) receptor antagonist, IL-10, and induced protein (IP)-10 [19]. Hypothetically, it is possible that these mediators from the periodontal lesions “spill over” into the systemic circulation and may attain concentrations that are sufficient to have their effects on the organs and tissues located distally from the oral cavity, provided their bioactivity remains preserved [20]. Eventually, other organs including the liver may be affected by these inflammatory mediators, thereby leading to the induction of an acute-phase response, which could influence more organs in the body including cardiovascular system. Promotion of cytokine production and upregulation of adhesion molecules result in the initiation and acceleration of inflammatory changes that ultimately induce atheroma in the endothelium, and this process is localized to the inner layer of arteries and disturb the blood flow [21]. Additionally, periodontal pathogens cause bacteremia that enter into endothelial cells, cause dysfunction, and induce a pro-atherogenic response in endothelial cells [22,23]. These deposits in arteries may vary from small to large size, which leads toward ischemia of the heart and results in thrombosis and infarction of blood vessels [21,24].

Oral and systemic disease connections have been a constant issue of discussion in scientific literature as “infections in the mouth can cause damage elsewhere in the body”, and two hundred possible associations between oral and systemic conditions are reported [25]. The relationship between poor oral health mainly due to periodontal disease and tooth loss and increased risk of CVDs, pulmonary diseases, diabetes, and pregnancy outcomes has been explained in the literature. [26] These chronic systemic conditions and oral diseases share many common risk factors of heredity, age, gender, education, sedentary lifestyle, smoking, diet, and obesity [27].

Additional research is warranted to better understand the relationship of PD and CVD to find out whether oral health assessment and management of PD could improve oral and general health and quality of life and be of relevance in the management of patients with CVD. This review aims to explore the current research to appreciate the plausible biological mechanism that links periodontitis with CVD, especially focusing on the emerging field of epigenetics. In this review, we have explored different frontiers in epigenetics and observe its implications in PD and CVD associations.

## 2. Epigenetics

Epigenetics is the study of mitotically and meiotically heritable changes in gene expression that are not dependent on DNA sequence, and the epigenome is the overall epigenetic state of an organism [28,29]. Epigenetics is on a continual rise for explaining the particular workings through which certain environmental factors were acting intermediately, having an effect on the gene expression without any alterations on the underlying genetic sequences [30]. Epigenetics, a term contrived by Conrad H. Waddington in 1942, was meant to bridge the gaps between genetics, growth, and differentiation, encompassing the “causal mechanisms” through which genes resulted in many and differing phenotypes [20,21,22,23,24,25,26,27,28,29,30,31,32]. Epigenetic mechanisms and modifications, defined as apparent DNA alterations that are heritable, entail environmental factors’ effect on modifying the gene expression without any change on the underlying DNA sequence [33]. In consequence, the changes are not encoded in the DNA molecules, but through subsequent chemical alterations, epigenetics leads to thorough remodeling of chromatin resulting in activation or suppression of gene expression [31]. Even though a considerable amount of literature is present underlining the consequences of environmental effect on the gene, little is known about the occurrence of an epigenetic eventuality concerning inflammatory pathways and gene expression phenotypes. As nature has it, all the cells present within the body mainly have the same genotype. Still, phenotypic expressions are different within these cells, which to some extents are the product of variations present in the epigenome, mainly due to environmental influences [30].

The environmental determinants, known and theorized, responsible for driving the epigenetic mechanisms forward include but are not limited to external factors such as life style, exposure to heavy metals such as mercury, radiations such as ultraviolet (UV), smoking, and infectious agents such as *Helicobacter pylori* [33,34,35]. These modifications may lead to single nucleotide polymorphism, which is incredibly responsive to changes in external environmental stimuli, thus orienting the expression of genes along with it [33].

A number of epigenetic processes have been identified in the literature and discussed in the below headings specifically periodontal diseases and cardiovascular diseases. Some of the essential mechanisms include DNA methylation, post-transcriptional histone modifications, including methylation, acetylation, ubiquitylation, sumoylation, and phosphorylation, which also affect the structure of chromatin and RNA-associated gene silencing (micro-RNA; miRNA) [34,36]. Regarding this, Benakenakare et al. [32], in their review, outlined the three major pathways through which epigenetic mechanisms work; the initial pathway is through the effect the external environmental factors have on the human body cells, which can result in alterations at the DNA level, known as the Epigenator. Following this are the non-coding RNAs known as the Epigenetic Initiator and Epigenetic Maintainer, which are responsible for sustaining these changes through generations by way of inheritance.

The process of DNA methylation and histone modification has been deemed the most common of the epigenetic processes in the medical literature taking place in human cells [36,37]. DNA methylation started with the help of DNA methyltransferases [33] and involves a covalent transfer of a methyl group (CH_3_) at the cytosine residues present within the cytosine–phosphate–guanine (CpG) dinucleotides found at the promoter region of the specific gene [32,38]. It is also the most scrutinized epigenetic mechanism in the literature in relation to cancer development and progress. DNA methylation, which occurs at CpG-rich islands in the DNA molecule comprises approximately 50% of the human genetic code. Additionally, the CpG-rich islands of the DNA are ordinarily unmethylated in normal human body tissues [32]. As a result, the consequent hypomethylation or hypermethylation of the promoter regions of associated genes leads to transcriptional activation or silencing, concluding with gene expression or loss of expression, respectively [37,38].

Histone modifications include the process of acetylation or deacetylation through enzymatic action, which results in chromatin remodeling that then influences gene expression [34,39]. The genetic material within humans is structured as a DNA helix packed up in the cell nucleus in the form of chromatin [31]. The chromatin’s basic unit comprises a nucleosome that is essentially DNA packaged around two copies each of H2A, H2B, H3, and H4, also known as the histone complex. These histones, in turn, have the ability to undergo post-translational histone modifications at their unstructured N-terminal tails through enzymatic actions of histone acetyltransferases (HATs) or histone deacetylases (HDACs) [36,40].

Epigenetics has, as a matter of fact, been used to explain the inexplainable genetic conundrums occurring in the fields of medicine and, more recently, dentistry as well. For example, some diseases to have suspected potential epigenetic mechanisms in their pathogenesis include cancer, depression, asthma, chronic obstructive pulmonary disease (COPD), and major psychosis, among others [30]. Even in the dental field, literature has shown the role of epigenetics and the potential gene therapy has in the case of oral cancers, infectious diseases, and autoimmune disorders [41,42].

## 3. Epigenetics and Periodontal Disease

Periodontal disease entails a pathological process that involves the whole of the tooth-supporting structures of the tooth, known as the periodontium; the gingiva, alveolar bone, cementum, and periodontal ligament (PDL) [43]. Periodontal disease, a significant global public health problem, ranges in a global disease burden from 20% to 70% [35,43]. Periodontitis has a multifactorial etiology including malocclusion, poor dental hygiene and excessive plaque and calculus formation, bruxism, poor tooth fillings, and ill-fitting prosthetics [31,35,37].

**Figure 1:**
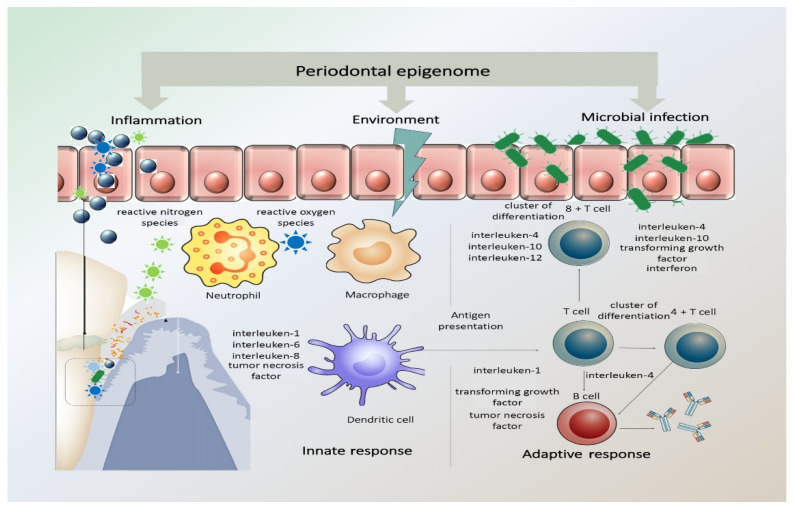
Periodontal epigenome in response to microbial infection, environmental stimuli, and inflammation. The periodontal inflammatory response has both protective and destructive elements that pathogens may alter. The innate inflammatory response relies on the recognition of microbial pathogens and is mediated by neutrophils, tissue macrophages, subepithelial dendritic cells, natural killer cells, and monocytes. The activated immune cells produce and release reactive oxygen species (ROS) and reactive nitrogen species (RNS) in response to infection. This acute inflammatory response turns to a chronic stage when the antigen-presenting cells become involved and present the antigens/microorganisms to immunocompetent cells expanding the antibody-secreting plasma cell population. (Adapted with permission from Wiley Publisher).

As is apparent in the literature, the effect of genetics and its impact of periodontitis pathogenesis has been a source of concern and research [44]. DNA methylation and histone modifications at the level of genetics and epigenetics have been studied, with particular attention to inflammation-related genes and associated immune system reactions through cytokines and chemokines [32,37]. It has also been postulated that the corresponding cytokines can also influence accompanying epigenetic changes within cells, thus modifying gene expression. The presence of chronic inflammation, such as in chronic periodontitis, coupled with the constant presence of Gram-negative bacteria due to poor oral hygiene, can in fact promote DNA methylation [37]. In Figure 1, representation of periodontal epigenome, how it responds to microbial infection, environmental stimuli, and inflammation is illustrated.

Periodontitis and periodontal disease construe significant destruction of the tooth-supporting structures and loss of bone with eventual loss of teeth themselves. Epigenetic mechanisms in turn regulate the effect of cytokines, such as interleukin-1 (IL-1) and IL-6, which are responsible for the progression of periodontitis [39]. In their study, Oliveira et al. [45] reported significant methylation patterns of the IL-8 gene promoter region in smokers with chronic periodontitis compared with healthy non-smoker counterparts. Smoking has also been deemed a significant environmental risk factor in the development and progress of periodontitis, causing persistent and everlasting hypermethylation and hypomethylation changes in the DNA. Alteration of the promoter region of the protein-coding gene prostaglandin-endoperoxide synthase 2 (PTGS2) has also been shown in the event of chronic periodontitis [46]. The PTGS2 gene is the encoding gene for the COX-2 enzyme responsible for producing prostaglandins, which cause inflammation and associated pain in the area. Understandably, the inhibition of the said COX-2 enzyme by administering COX-2 inhibitors reduced the symptoms found in periodontitis patients. However, expression of COX-2 enzymes in the inflamed periodontal tissues in chronic periodontitis patients was technically lower, and the gene promoter was found to be hypermethylated [32]. Moreover, certain lipopolysaccharides (LPS) associated with the periodontal pathogens can cause epigenetic mitigations, which are commonly found in periodontitis and periodontal disease [33].

Of the many signs and symptoms related to periodontal disease, one of extreme significance includes alveolar bone loss leading to the subsequent loss of teeth. Cantley et al. [47] in their experimental study on mice induced periodontitis by inoculating *Porphyromonas gingivalis* into their oral cavities. A novel histone deacetylase inhibitor (HDACi), 1179.4b, was then administered to the one of the experimental groups of mice, which showed a significant reduction in bone loss indicating that suppressing the associated epigenetic mechanism is responsible for osteoclast-mediated bone loss. Similarly, *Treponema denticola* also acts through various epigenetic mechanisms leading to a consequent loss of tooth-supporting alveolar bone frequently seen in periodontal disease [31,48].

As the first line of defense in the oral cavity is the oral epithelial cells against foreign bodies including bacterial/viral pathogens, specific epigenetic changes can be induced by the pathogens resulting in histone acetylation and demethylation. Furthermore, the activation of toll-like receptors (TLRs) and pathogen-recognition receptors (PRRs) can substantially stimulate modifications of histones in the epithelial cells of the oral cavity [31]. Loss of periodontal ligament attachment is also seen in periodontal disease, which is affected by dysregulated cytokine immune response that has been tampered with through epigenetic pathways [31]. Changes in the methylation patterns of CCL25 and IL17C, which are the cytokines responsive to bacterial invasion, and the increased gene expression of the TH17 T helper cell leads to the loss of periodontal attachment frequently observed in periodontitis [49,50].

## 4. Epigenetics and Cardiovascular Diseases (CVD)

Epigenetics have opened a new world to understand and manage human diseases, including CVDs [51]. Genetic and environmental factors may lead to changes in several pathways that ultimately activate the development of a disease [52]. Cardiovascular diseases contribute from a multifactorial and complex interplay of genetic, environmental, and lifestyle factors [53]. Epigenetic changes can modify gene transcription by altering the accessibility of gene transcription machinery, and these changes are predisposed by factors of nutrition, inflammation, sex, age, and lifestyle changes [54]. Chronic exposure to novel lifestyle factors coinciding with disease mechanisms involving chronic low-grade systemic inflammation affects vascular health [2]. Epigenetic modifications and miRNA may play a crucial role in the development of pathological conditions such as CVDs. Mechanisms underlying the complex pathophysiology that leads to CVDs are of great interest but still far from clear [51]. Epigenetics involves changes in gene expression due to chromatin adjustments that change the accessibility of DNA without changing its sequence, leading to silencing or downregulation/upregulation of gene expression [55].

Epigenetic modifications involved in the initiation and progression of atherosclerosis play an essential role in plaque development and vulnerability [56]. Accumulation of cholesterol in the walls of large- and medium-sized arteries, the accumulation of extracellular matrix and lipids, and smooth muscle cell proliferation leads to the infiltration of immune cells (mostly macrophages) and endothelial dysfunction, forming a plaque and, eventually, developing into acute cardiovascular events, such as MI, peripheral vascular disease, aneurysms, and stroke [57]. Inflammatory pathways are set as a target to improve treatment of patients, and the emergence of epigenetic modifiers as anti-inflammatory agents in many chronic inflammatory disorders such as CVD may benefit from the evaluation of such compounds [58,59]. 

Methylation is also suggested as an indicator of MI [60]. Abundant research has focused on miRNAs as novel biomarkers for MI. MiR-1 levels have been analyzed in plasma from patients with AMI and found to be significantly elevated, but decreased to normal levels with medication [61]. DNA methylation is associated with gene silencing, while histone modifications can either result in gene activation or silencing. In addition to DNA methylation, different histone modifications set the histone code and regulate the interaction and function of transcriptions factors. As such, a large number of histone-modifying enzymes regulate myeloid cell differentiation, macrophage polarization, and the ensuing macrophage phenotype [59]. The combination of both acetylation and methylation modifications of histone tails determine the histone code of enhancers and promoters and thereby control gene transcription or repression [62]. 

## 5. Conclusions

Oral inflammatory diseases have been shown to contribute to disease states and inflammatory pathology at sites distant from the oral cavity. Studies highlight how the inflammatory status of the oral cavity can have a profound impact on systemic health. Periodontitis, an inflammatory disease of the oral mucosa, is epidemiologically associated with other chronic inflammation-driven disorders, including cardio-metabolic, neurodegenerative, and autoimmune diseases and cancer.

The potential causal link between periodontitis and its comorbidities is further strengthened by recent experimental animal studies establishing biologically plausible and clinically consistent mechanisms whereby periodontitis could initiate or aggravate a comorbid condition. This multi-faceted “mechanistic causality” aspect of the link between periodontitis and comorbidities is the focus of this review. The understanding how certain extra-oral pathologies are affected by disseminated periodontal pathogens and periodontitis-associated systemic inflammation, including adaptation of bone marrow hematopoietic progenitors, may provide new therapeutic options to reduce the risk of periodontitis-associated comorbidities. 

Emerging evidence from interventional studies indicates that local treatment of periodontitis ameliorates surrogate markers of comorbid conditions. In recent years, genetic medicine has started a new era of epigenetics to overcome human diseases with various new methodology [63]. The current century has recognized the role of DNA in biology and medicine and viewed DNA as the “book of life”. Phenotypes depend on specific combinations of genome composition, epigenetic components, and environmental inputs. This has allowed the biomedical community to test the relevance of epigenetic features in specific disease functions and use them as prognostic and diagnostic markers [16,23,64,65]. 

A most recent prospective study conducted over 13 years has demonstrated that severe periodontitis is associated with an increased incidence of CHD independent of established risk factors [66]. Risk factors for PD and CVD are determined both genetically and behaviorally. Environmental factors are important in determining an individual’s predisposition to develop major cardiovascular risk factors through epigenetic modifications and the identification of the epigenetic mechanisms that participate in disease development [62]. A better understanding of the exact regulatory mechanisms of epigenetic pathways driving the inflammation is slowly emerging and will aid in developing novel tools for treatment of disease. It may possibly also aid in better diagnosis and stratification of patients based on their epigenetic profile, showing potential predisposed states for disease. Macrophages, key cells in inflammation advancement or resolution, play an important role in hemostatic process, and epigenetic mechanisms modulate signals during macrophage polarization. Activated macrophages polarize towards various functional phenotypes [67]. Future targeting of specific epigenetic pathways in cardiovascular disease may, thus, offer exciting novel approaches for the treatment of disease [54].

## References

[1] Ahmed S.H., Meyer H.E., Kjøllesdal M.K., Marjerrison N., Mdala I., Htet A.S., Bjertness E., Madar A.A. (2019). The prevalence of selected risk factors for non-communicable diseases in Hargeisa, Somaliland: A cross-sectional study. BMC Public Health.

[2] Lechner K., von Schacky C., McKenzie A.L., Worm N., Nixdorff U., Lechner B., Kränkel N., Halle M., Krauss R.M., Scherr J. (2020). Lifestyle factors and high-risk atherosclerosis: Pathways and mechanisms beyond traditional risk factors. Eur. J. Prev. Cardiol..

[3] Crea F. (2020). Interaction between predisposing genes and environmental risk factors in cardiovascular disease: How prevention can counteract this salty combination. Eur. Heart J..

[4] Buttar H.S., Li T., Ravi N. (2005). Prevention of cardiovascular diseases: Role of exercise, dietary interventions, obesity and smoking cessation. Exp. Clin. Cardiol..

[5] Yusuf S., Joseph P., Rangarajan S., Islam S., Mente A., Hystad P., Brauer M., Kutty V.R., Gupta R., Wielgosz A. (2020). Modifiable risk factors, cardiovascular disease, and mortality in 155 722 individuals from 21 high-income, middle-income, and low-income countries (PURE): A prospective cohort study. Lancet.

[6] Könönen E., Gursoy M., Gursoy U.K. (2019). Periodontitis: A Multifaceted Disease of Tooth-Supporting Tissues. J. Clin. Med..

[7] Nazir M.A. (2017). Prevalence of periodontal disease, its association with systemic diseases and prevention. Int. J. Health Sci..

[8] Lamont R.J., Koo H., Hajishengallis G. (2018). The oral microbiota: Dynamic communities and host interactions. Nat. Rev. Genet..

[9] Cekici A., Kantarci A., Hasturk H., Van Dyke T.E. (2014). Inflammatory and immune pathways in the pathogenesis of periodontal disease. Periodontology 2000.

[10] Konkel J.E., O’Boyle C., Krishnan S. (2019). Distal Consequences of Oral Inflammation. Front. Immunol..

[11] Liccardo D., Cannavo A., Spagnuolo G., Ferrara N., Cittadini A., Rengo C., Rengo G. (2019). Periodontal Disease: A Risk Factor for Diabetes and Cardiovascular Disease. Int. J. Mol. Sci..

[12] Hajishengallis G., Chavakis T. (2021). Local and systemic mechanisms linking periodontal disease and inflammatory comorbidities. Nat. Rev. Immunol..

[13] Dhadse P., Gattani D., Mishra R. (2010). The link between periodontal disease and cardiovascular disease: How far we have come in last two decades?. J. Indian Soc. Periodontol..

[14] Janket S.-J., Javaheri H., Ackerson L., Ayilavarapu S., Meurman J. (2015). Oral Infections, Metabolic Inflammation, Genetics, and Cardiometabolic Diseases. J. Dent. Res..

[15] Lopez-Candales A., Burgos P.M.H., Hernandez-Suarez D.F., Harris D. (2017). Linking Chronic Inflammation with Cardiovascular Disease: From Normal Aging to the Metabolic Syndrome. South Pac. J. Nat. Appl. Sci..

[16] Rehman S.A., Khurshid Z., Niazi F.H., Naseem M., Al Waddani H., Sahibzada H.A., Khan R.S. (2017). Role of Salivary Biomarkers in Detection of Cardiovascular Diseases (CVD). Proteomes.

[17] Carrizales-Sepúlveda E.F., Ordaz-Farías A., Vera-Pineda R., Flores-Ramírez R. (2018). Periodontal Disease, Systemic Inflammation and the Risk of Cardiovascular Disease. Hear. Lung Circ..

[18] Schenkein H.A., Loos B.G. (2013). Inflammatory mechanisms linking periodontal diseases to cardiovascular diseases. J. Clin. Periodontol..

[19] Ramadan D.E., Hariyani N., Indrawati R., Ridwan R.D., Diyatri I. (2020). Cytokines and Chemokines in Periodontitis. Eur. J. Dent..

[20] Makkar H., Reynolds M.A., Wadhawan A., Dagdag A., Merchant A.T., Postolache T.T. (2018). Periodontal, metabolic, and cardiovascular disease: Exploring the role of inflammation and mental health. Pteridines.

[21] Teles R., Wang C.-Y. (2011). Mechanisms involved in the association between peridontal diseases and cardiovascular disease. Oral Dis..

[22] Ahmad P., Arshad A.I., Della Bella E., Khurshid Z., Stoddart M. (2020). Systemic Manifestations of the Periodontal Disease: A Bibliometric Review. Molecules.

[23] Bibi T., Khurshid Z., Rehman A., Imran E., Srivastava K., Shrivastava D. (2021). Gingival Crevicular Fluid (GCF): A Diagnostic Tool for the Detection of Periodontal Health and Diseases. Molecules.

[24] Forner L., Larsen T., Kilian M., Holmstrup P. (2006). Incidence of bacteremia after chewing, tooth brushing and scaling in individuals with periodontal inflammation. J. Clin. Periodontol..

[25] Bokhari S., Khan A. (2009). Growing burden of noncommunicable diseases: The contributory role of oral diseases, Eastern Mediterranean Region perspective. East. Mediterr. Health J..

[26] Joshipura K., Ritchie C., Douglass C. (2000). Strength of evidence linking oral conditions and systemic disease. Compend. Contin. Educ. Dent. (Jamesburg NJ 1995). Suppl..

[27] Page R.C. (1998). The Pathobiology of Periodontal Diseases May Affect Systemic Diseases: Inversion of a Paradigm. Ann. Periodontol..

[28] Ramesh S., Neelakantan P. (2014). Systemic diseases and oral health. Indian J. Med Spec..

[29] Bayarsaihan D. (2010). Epigenetic Mechanisms in Inflammation. J. Dent. Res..

[30] Handel A., Ebers G.C., Ramagopalan S.V. (2010). Epigenetics: Molecular mechanisms and implications for disease. Trends Mol. Med..

[31] Larsson L. (2017). Current Concepts of Epigenetics and Its Role in Periodontitis. Curr. Oral Health Rep..

[32] Benakanakere M.R., Finoti L., Palioto D.B., Teixeira H.S., Kinane D.F. (2019). Epigenetics, Inflammation, and Periodontal Disease. Curr. Oral Health Rep..

[33] Barros S.P., Hefni E., Nepomuceno R., Offenbacher S., North K. (2018). Targeting epigenetic mechanisms in periodontal diseases. Periodontology 2000.

[34] Weinhold B. (2006). Epigenetics: The Science of Change. Environ. Health Perspect..

[35] Rygiel K., Jośko-Ochojska J. (2018). Diseases of the oral cavity in light of the newest epigenetic research: Possible implications for stomatology. Adv. Clin. Exp. Med..

[36] Srinivasan P.C. (2016). The emerging role of epigenetics in the pathogenesis of periodontitis-A review. S. Afr. Dent. J..

[37] Gomez R.S., Dutra W.O., Moreira P.R. (2009). Epigenetics and periodontal disease: Future perspectives. Inflamm. Res..

[38] Rao S.R., Lavu V., Venkatesan V. (2015). The epigenetic paradigm in periodontitis pathogenesis. J. Indian Soc. Periodontol..

[39] Chaurasia A. (2017). Epigenetics in Periodontal Diseases. J. Clin. Epigenetics.

[40] Sanders V.M. (2006). Epigenetic regulation of Th1 and Th2 cell development. Brain Behav. Immun..

[41] Khurshid Z. (2018). Future of Oral Proteomics. J. Oral Res..

[42] Siddique N., Raza H., Ahmed S., Khurshid Z., Zafar M.S. (2016). Gene Therapy: A Paradigm Shift in Dentistry. Genes.

[43] Nazir M., Al-Ansari A., Al-Khalifa K., Alhareky M., Gaffar B., Almas K. (2020). Global Prevalence of Periodontal Disease and Lack of Its Surveillance. Sci. World J..

[44] Kinane D.F., Shiba H., Hart T.C. (2005). The genetic basis of periodontitis. Periodontology 2000.

[45] de Oliveira N.F.P., Damm G.R., Andia D., Salmon C., Nociti F.H., Line S.R.P., De Souza A.P. (2009). DNA methylation status of theIL8gene promoter in oral cells of smokers and non-smokers with chronic periodontitis. J. Clin. Periodontol..

[46] Zhang S., Barros S.P., Niculescu M.D., Moretti A.J., Preisser J.S., Offenbacher S. (2010). Alteration of PTGS2 Promoter Methylation in Chronic Periodontitis. J. Dent. Res..

[47] Cantley M.D., Bartold P.M., Marino V., Fairlie D., Le G.T., Lucke A., Haynes D.R. (2011). Histone deacetylase inhibitors and periodontal bone loss. J. Periodontal Res..

[48] Najeeb S., Khurshid Z., Agwan M.A.S., Ansari S.A., Zafar M.S., Matinlinna J.P. (2017). Regenerative Potential of Platelet Rich Fibrin (PRF) for Curing Intrabony Periodontal Defects: A Systematic Review of Clinical Studies. Tissue Eng. Regen. Med..

[49] Schulz S., Immel U.D., Just L., Schaller H.-G., Gläser C., Reichert S. (2016). Epigenetic characteristics in inflammatory candidate genes in aggressive periodontitis. Hum. Immunol..

[50] Khurshid Z., Zohaib S., Najeeb S., Zafar M.S., Rehman R., Rehman I.U. (2016). Advances of Proteomic Sciences in Dentistry. Int. J. Mol. Sci..

[51] Soler-Botija C., Gálvez-Montón C., Bayés-Genís A. (2019). Epigenetic Biomarkers in Cardiovascular Diseases. Front. Genet..

[52] Franceschini N., Le T.H. (2014). Genetics of hypertension: Discoveries from the bench to human populations. Am. J. Physiol. Physiol..

[53] Avijeeta A. (2019). Periodontitis and Cardiovascular Diseases: The Nexus. J. Med Sci. Clin. Res..

[54] Gallardo-Escribano C., Buonaiuto V., Ruiz-Moreno M.I., Vargas-Candela A., Vilches-Perez A., Benitez-Porres J., Romance-Garcia A.R., Ruiz-Moreno A., Gomez-Huelgas R., Bernal-Lopez M.R. (2020). Epigenetic approach in obesity: DNA methylation in a prepubertal population which underwent a lifestyle modification. Clin. Epigenetics.

[55] Baccarelli A., Rienstra M., Benjamin E. (2010). Cardiovascular Epigenetics. Circ. Cardiovasc. Genet..

[56] Xu S., Pelisek J., Jin Z.G. (2018). Atherosclerosis Is an Epigenetic Disease. Trends Endocrinol. Metab..

[57] Wissler R.W. (1991). Update on the pathogenesis of atherosclerosis. Am. J. Med..

[58] Hoeksema M.A., Stöger J.L., De Winther M.P.J. (2012). Molecular Pathways Regulating Macrophage Polarization: Implications for Atherosclerosis. Curr. Atheroscler. Rep..

[59] Neele A.E., Bossche J.V.D., Hoeksema M.A., de Winther M.P. (2015). Epigenetic pathways in macrophages emerge as novel targets in atherosclerosis. Eur. J. Pharmacol..

[60] Talens R.P., Jukema J.W., Trompet S., Kremer D., Westendorp R.G.J., Lumey L.H., Sattar N., Putter H., E Slagboom P., Heijmans B.T. (2011). Hypermethylation at loci sensitive to the prenatal environment is associated with increased incidence of myocardial infarction. Int. J. Epidemiol..

[61] Ai J., Zhang R., Li Y., Pu J., Lu Y., Jiao J., Li K., Yu B., Li Z., Wang R. (2010). Circulating microRNA-1 as a potential novel biomarker for acute myocardial infarction. Biochem. Biophys. Res. Commun..

[62] Bossche J.V.D., Neele A.E., Hoeksema M., de Winther M. (2014). Macrophage polarization. Curr. Opin. Lipidol..

[63] Moosavi A., Ardekani A.M. (2016). Role of Epigenetics in Biology and Human Diseases. Iran. Biomed. J..

[64] Khurshid Z., Mali M., Naseem M., Najeeb S., Zafar M.S. (2017). Human Gingival Crevicular Fluids (GCF) Proteomics: An Overview. Dent. J..

[65] Cavalli G., Heard E. (2019). Advances in epigenetics link genetics to the environment and disease. Nat. Cell Biol..

[66] Tiensripojamarn N., Lertpimonchai A., Tavedhikul K., Udomsak A., Vathesatogkit P., Sritara P., Charatkulangkun O. (2021). Periodontitis is associated with cardiovascular diseases: A 13-year study. J. Clin. Periodontol..

[67] Xuan D., Han Q., Tu Q., Zhang L., Yu L., Murry D., Tu T., Tang Y., Lian J.B., Stein G.S. (2015). Epigenetic Modulation in Periodontitis: Interaction of Adiponectin and JMJD3-IRF4 Axis in Macrophages. J. Cell. Physiol..

